# Quality Improvement Methodology Optimizes Infliximab Levels in Pediatric Patients with Inflammatory Bowel Disease

**DOI:** 10.1097/pq9.0000000000000400

**Published:** 2021-05-05

**Authors:** Jennifer Hellmann, Renee K. Etter, Lee A. Denson, Phillip Minar, Denise Hill, Dana M. Dykes, Michael J. Rosen

**Affiliations:** From the * Division of Gastroenterology, Hepatology, and Nutrition, Cincinnati Children’s Hospital Medical Center, Cincinnati, Ohio; † Department of Pediatrics, University of Cincinnati College of Medicine, Cincinnati, Ohio; ‡ Department of Information Services, Cincinnati Children’s Hospital Medical Center, Cincinnati, Ohio; § GI Care for Kids, Atlanta, Ga.

## Abstract

Achieving and maintaining target serum trough infliximab levels improves outcomes in children and young adults with inflammatory bowel disease. Our goal was to improve adherence to an infliximab therapy guideline. The primary aim was to increase the percentage of patients with infliximab levels ≥5 μg/mL and results checked in the last 12 months from 73% to ≥80% from July 2017 to January 2018.

**Methods:**

We participated in Intermediate Improvement Science Series, a course at Cincinnati Children’s Hospital Medical Center designed to catalyze change using quality improvement methodology. We implemented interventions through plan-do-study-act cycles. Our outcome measure was balanced by 2 process measures to determine what actions impacted improvement. These measures included the percentage of infusion plans revised in response to a drug level <5 μg/mL and the proportion of plans for which a follow-up drug level was ordered.

**Results:**

We increased the percentage of infusion plans revised before the next infusion from 63% to 87% and the percentage of plans that had an appropriate drug level recheck from 61% to 83% from July 2017 to January 2018. We increased the percentage of patients with an infliximab level >5 μg/mL, and results checked in the last 12 months, from 73% to 80%.

**Conclusions:**

Quality improvement methodology was effective in improving provider adherence to infliximab therapeutic drug monitoring guidelines. Improvement in adherence to guidelines directly improved the percentage of patients achieving target infliximab levels at any time during infliximab therapy.

## Abstract 2

Supplemental Digital Content is available in the text.

## INTRODUCTION

Antitumor necrosis factor (anti-TNF) agents such as infliximab are useful in the treatment of moderate-to-severe Crohn’s disease and ulcerative colitis in children.^1,2^ However, secondary loss of response has been reported in approximately one-third of patients due to low drug levels and the development of antidrug antibodies.^3–5^ Proactive therapeutic drug monitoring, defined as drug level monitoring in patients with a clinical response/remission with dose adjustments made to target drug trough, in patients receiving anti-TNF therapy for inflammatory bowel disease (IBD) has been shown to reduce the risk of treatment failure and antidrug antibody development.^6–11^

In 2014, Cincinnati Children’s Hospital Medical Center (CCHMC) began an initiative to standardize clinical practice across disciplines. Within the Division of Gastroenterology, Hepatology, and Nutrition (GI division), the focus was on personalized, cost-effective infliximab use in patients with IBD. To this end, an infliximab care algorithm was created and further modified in 2016. Key components of this guideline included the recommendation to check an infliximab level before the fourth dose and at least annually thereafter, a goal trough of ≥5 μg/mL for all patients, a recommendation to alter either infliximab dose or interval for those with a level <5 μg/mL, and guidance on initiation of an immunomodulator. Infliximab drug levels were assessed using a drug-tolerant electrochemiluminescence immunoassay (ECLIA; LabCorp, Burlington, N.C.). However, many providers were initially nonadherent to the new guideline, and approximately 25% of patients receiving infliximab at CCHMC had subtherapeutic drug levels in 2016. To address the problem of guideline nonadherence, and, in turn, subtherapeutic drug levels, we implemented a formal quality improvement (QI) initiative starting in July 2017.

We utilized the “Model for Improvement,” which focuses on building teams, setting aims, selecting, testing, and implementing changes using plan-do-study-act (PDSA) cycles to catalyze a positive change.^12^ This methodology has resulted in local high-impact improvements such as better adherence to pediatric pneumonia treatment guidelines and improvements in the hospital discharge process.^13,14^ Our project’s primary aim was to increase the percentage of patients with infliximab drug levels ≥5 μg/mL, and results checked in the last 12 months from 73% in July 2017 to ≥80% in January 2018. This measure encompassed both the target trough level and the minimum goal of annual therapeutic drug monitoring. By improving adherence to our infliximab care algorithm, we sought to impact our global aim of improving sustained clinical remission rates in children and young adults with IBD.

## METHODS

### Setting

Our team performed this project at a large, urban academic hospital and associated suburban satellite locations that serve as a regional and national referral center. The Schubert Martin Inflammatory Bowel Disease Center operates within the GI Division at CCHMC, and, at the time of the project, was staffed by 3–4 physicians, a team of nurses, and a nurse program administrator. There were 26 additional clinical gastroenterology providers within the division in July 2017. The center sees an estimated 900 IBD patients annually, of which approximately 300 receive infliximab infusions at one of our affiliated sites.

### Inclusion/Exclusion Criteria and Definitions

This project included patients with a diagnosis of Crohn’s disease, ulcerative colitis, and IBD-unclassified. Patients had to receive infusions through CCHMC, including our infusion center, inpatient hospital, satellite hospital, and home care services to be included in the analysis. This work did not include patients receiving infusions at external infusion centers, including outside hospitals, external home care companies, and freestanding infusion centers. This criterion excluded approximately 70 (23%) of our patients receiving infliximab. Our work included all patients with drug levels during the maintenance phase of therapy (beyond the third infusion).

We collected data on drug levels and responses to drug levels from our electronic medical record (EMR) (Epic Systems Corporation, Verona, Wis.). Sustained clinical remission data is routinely reported every month through ImproveCareNow (ICN), an international QI collaborative for pediatric IBD providers, patients, and families. Per ICN, sustained clinical remission is defined as a physician global assessment (PGA) of quiescent for each clinic visit with no interim relapses in the past 365 days. PGA includes an assessment of clinical symptoms, physical examination, and laboratory values.^15^ To be considered quiescent, patients must be asymptomatic and have no or transient laboratory abnormalities including hemoglobin, albumin, and inflammatory markers. Patients are included in the monthly reported measure if they had a visit within the past 13 months, are at least 477 days from diagnosis (excluding the first 3 months after diagnosis) and have been an established patient in the practice for at least 365 days.^15^ This study included only patients receiving infliximab in the analysis of change in sustained clinical remission.

### Process Mapping

We first generated an ideal process for proactive therapeutic drug monitoring using a high-level process map (**figure, Supplemental Digital Content 1**, which describes high-level ideal process map of proactive therapeutic drug monitoring: this process map was created based on the infliximab care algorithm generated in 2014. This process map begins with initiation of infliximab via order set that includes a drug level at the fourth dose. If a low drug level results, a change is recommended and the process repeats until drug levels are >5 μg/mL, http://links.lww.com/PQ9/A251) based on our infliximab care algorithm. The process encompassed drug ordering, drug trough ordering at the fourth dose (first maintenance dose at approximately 14 wks), provider notification of drug concentrations <5 μg/mL, and the provider’s treatment decision in response to the level. Providers repeated this process until a drug level of *≥*5 μg/mL was achieved.

**Fig. 1.**
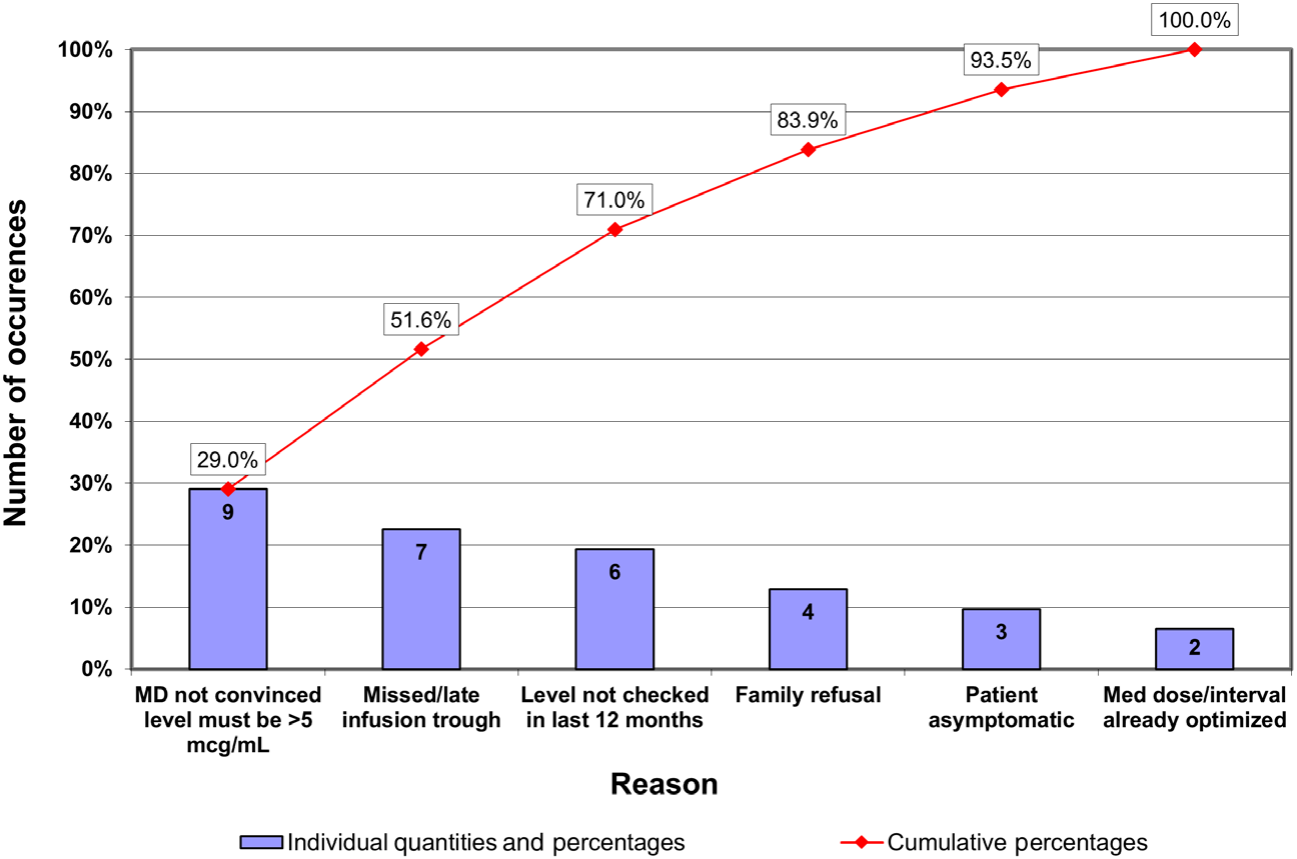
Pareto chart of reasons why a change was not made with a level <5 μg/mL. This chart depicts the reasons why a change in infliximab dose or dosing interval was not made. The most common reason for the failure of the process was physician belief that a drug level *>*5 μg/mL was unnecessary.

Before beginning interventions, we gathered data on failures in this process. We met with the 26 providers within our GI division to determine the reasons for nonadherence to the infliximab care algorithm. We used this data to create a Pareto chart (Fig. 1) to illustrate the cause and frequency of nonadherence failures. The leading failure was providers’ opinions regarding the optimal trough level based on personal experience and prior evidence.

### Key Drivers and Interventions

**Fig. 2.**
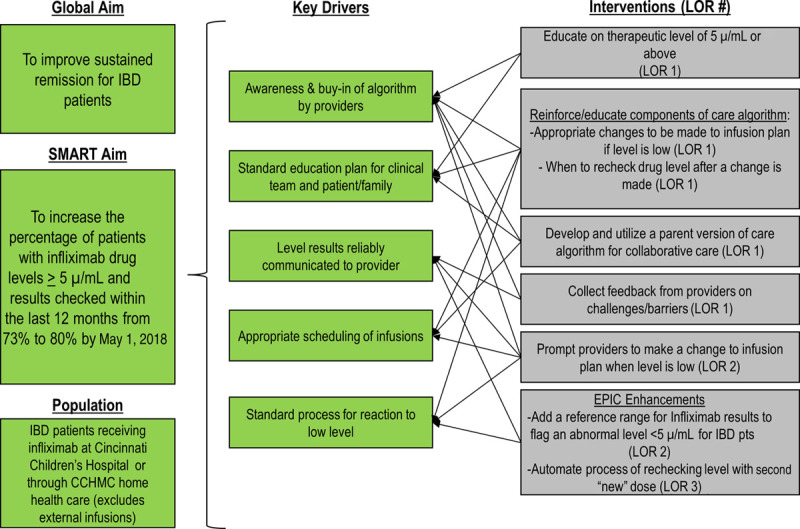
Therapeutic drug monitoring key driver diagram. The key driver diagram depicts our global aim, SMART aim, and the affected population. We identified 5 key drivers to impact our aims and identified interventions to impact the key drivers. Arrows are used to tie interventions to the key drivers they impact. LOR #, level of reliability number for any particular intervention, for example, LOR 1.

Once we established an ideal process and identified the causes of process failures, we created a key driver diagram, which included essential key drivers and interventions as our roadmap to drive improvement (Fig. 2). Using the model for improvement, we developed ideas for change into testable interventions directly related to the key drivers using the PDSA approach.^16^

As depicted in Figure 2, we also assigned interventions a level of reliability (LOR) with levels established from 1 to 3. These levels indicate the expected failure rates with level 1 primarily comprised employee and patient education and hard work with a typical expected failure rate of 1–2 out of 10. LOR 2 interventions typically result in <5 failures per 100 and are dependent on a built-in process in coordination with human effort, whereas LOR 3 interventions have an expected <5 failures per 1,000 opportunities and primarily rely on systems rather than sheer human effort.^17^

**Fig. 3.**
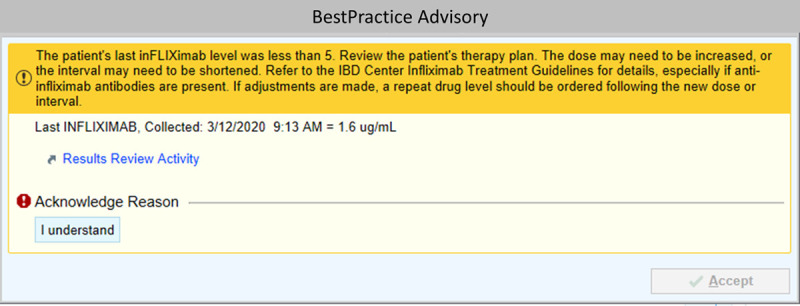
Best practice alert for an infliximab level <5 μg/mL in the last 365 days. This BPA fires upon opening a patient’s chart if the last documented infliximab level is <5 μg/mL. This alert fires only if the chart is opened by a gastroenterology attending, fellow or resident physician and only if there is an active gastroenterology infliximab infusion therapy plan. The provider receives this alert on a specific patient 1 time in 4 months to account for the time required to obtain a repeat drug level. ©2020 Epic Systems Corporation. Used with permission.

1. Awareness and buy-in of algorithm by providers. Physicians received education at faculty meetings, one-on-one meetings, and via email. We placed focus on the recommended infliximab therapy plan order revisions for patients with suboptimal levels and the concept of rechecking a trough before the second new infusion after a change. Provider feedback was continuously solicited and incorporated to ensure ongoing engagement by the key stakeholders.
2. Standard education plan for clinical team/patient/family. Nurses received education at staff meetings, one-on-one meetings, and via email. Patient and family input was used to design the parent algorithm (**figure, Supplemental Digital Content 2**, which describes infliximab care algorithm for parent education. This algorithm was created to provide to parents if their child was starting infliximab. The goal of the algorithm was to educate parents and patients on the expected interval of infliximab and reasons why a dose or infusion interval change may be recommended, http://links.lww.com/PQ9/A252). Nurses and physicians used this tool with patients and families to provide education and prompt questions about infliximab management.
3. Results reliably communicated to provider. Infliximab infusions are ordered through our EMR and physicians automatically receive notification of lab results via Epic. Additionally, each physician receives a previsit planning form in advance of each IBD patient visit. These forms note the most recent infliximab level. In addition to these standing processes, providers performed a weekly review on upcoming infusion patients whose last result trough level was <5 μg/mL. Our IBD nurse program administrator completed this review in our weekly previsit planning meeting. Providers who had adhered to the guideline received positive feedback via email. We contacted nonadherent providers via email with the recommended therapy plan order revisions.
4. Appropriate scheduling of infusions. This key driver was targeted as part of our physician and patient/family education intervention and automated prompts to change infusion interval. Nursing staff and administrative staff were key stakeholders to communicate the change during the interval and schedule the patient accordingly.
5. Standard process for reaction to low levels. Building on our education series, we sought to automate the reminder process through our EMR. We created Best Practice Alerts (BPA) within our EMR based on our process measure. The first alert triggered upon opening a chart, if the last infliximab level within 365 days was <5 μg/mL (Fig. 3). This BPA advised dose adjustment and level recheck in accordance with our care algorithm. The second alert fired when physicians revised the therapy plan but failed to order a repeat trough level. This intervention was designed to complement but eventually replace manual chart review and prompting by the IBD nurse program administrator to ensure a reliable process.

### Measures

A “SMART” aim that is specific, measurable, achievable, relevant and time-bound was created.^16^ Our aim is outlined in Figure 2. This measure encompassed our target trough level as well as our objective to check troughs at least annually. We did not include patients who did not have a drug level resulted within the past 12 months, but prompts were given to managing providers to order a drug level. Once they had a resulted level, patients were included. In the initial cohort of 125 patients, we initially excluded 4 (3%) patients due to lack of a drug level in the prior 12 months. Our SMART aim was considered an outcome measure because it directly related to the global aim to improve sustained clinical remission and would offer evidence that management changes were having an impact at the system level.^16^ Twelve months of baseline data were collected before initiation of the therapeutic drug monitoring QI initiative, ensuring we had stable baseline.

To coincide with our outcome measure, we developed balancing process measures for the outcome.^16^ The process measures were designed to focus on the steps taken to improve infliximab trough levels. If a patient’s trough was less than our target, the provider was expected to change the patient’s infusion plan based on the infliximab care algorithm. A successful therapy plan revision was 2-fold: a change made to the medication (dose or interval change) and a repeat drug trough ordered with the second new dose after the change.

### Analysis

We tracked both actions required for a successful therapy plan revision separately to visualize which action, if either, was driving change. We had minimal baseline data at the start of the therapeutic drug monitoring QI initiative in July 2017. The initial baseline identified in July 2017 for the percentage of infusion therapy plans appropriately changed was 63% based on data from May and July 2017. Our SMART aims were to increase the percentage of therapy plans appropriately changed in response to a low level from 63% to ≥80% and increase the percentage of follow-up drug levels ordered from 61% to ≥80% from July 2017 to January 2018. We predicted that proactive therapeutic drug monitoring would increase the percentage of trough levels in the therapeutic range and, in turn, improve sustained clinical remission rates for our population, based on existing literature.^7,10,18–20^

We analyzed both outcome and process measures using run charts. Statistically meaningful change on a run chart is represented by a shift, commonly defined as 8 points above or below the median.^21^ Statistical process control, a standard tool determining improvement, not due to natural variation in QI work, was used to depict and analyze change in sustained clinical remission in patients receiving infliximab.^22^ Standard rules for centerline changes were used; in this case, 8 points in a row above or below the centerline.

### Ethical Considerations

The CCHMC Institutional Review Board determined this project was quality or process improvement project and exempt from review.

## RESULTS

**Fig. 4.**
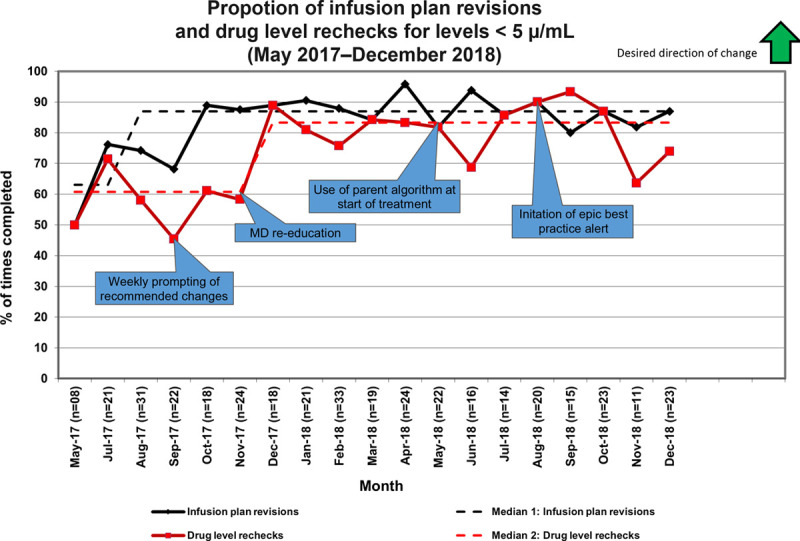
Run chart of proportion of infusion plan revisions and drug level rechecks for drug level <5 μg/mL. This run chart depicts our two process measures: the percentage of infusion plan revisions (dose change or dosing interval change) and the percentage of drug rechecks ordered after the dosing change. The medians of each measure are separately noted as median 1 and median 2, respectively. Data call-outs note interventions during the formal QI work.

By implementation of the ideal process and refinement based on multiple PDSA cycles, our measures improved. We increased the percentage of infusion plans revised before the next infusion from 63% to 87% (Fig. 4, median 1). The percentage of plans that had a drug level rechecked at the second new infusion also improved from 61% to 83% (Fig. 4, median 2). There was rapid improvement in therapy plan revisions but a lag in adopting the recommendation to order a follow-up drug level. Focused education on this issue and implementing a BPA recommending a follow-up drug level resulted in change.

**Fig. 5.**
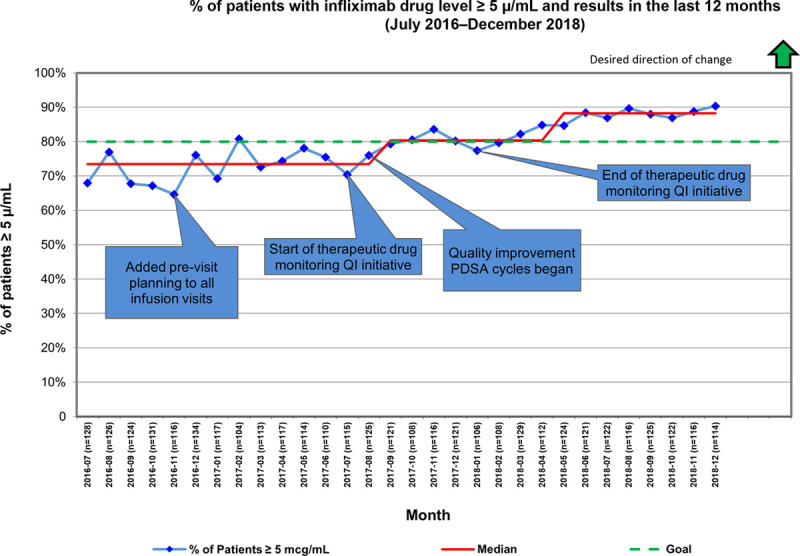
Percentage of patients with infliximab drug level *>*5 μg/mL and results in the last 12 months. This run chart depicts our outcome measure: the percentage of patients with a drug level at goal and checked within the last 12 months from July 2016 to December 2018. Baseline data were from July 2016 to the start of the therapeutic drug monitoring QI initiative in July 2017. Data call-outs note the beginning and end of this work.

Improved process measures were associated with improvement in our outcome measure. Each month, between 104 and 134 patients received an infusion in the prior month and met inclusion criteria for inclusion in the outcome measure. We observed an increase in patients’ percentage with an optimal level ≥5 μg/mL and results in the last 12 months from 73% to 80% between July 2017 and January 2018 (Fig. 5). Data tracking continued after the end of the therapeutic drug monitoring QI initiative. This outcome measure continued to improve with time, indicating that lasting practice change had taken root in the GI division. Our outcome measure further improved from 80% in January 2018 to 88% in January 2019.

**Fig. 6.**
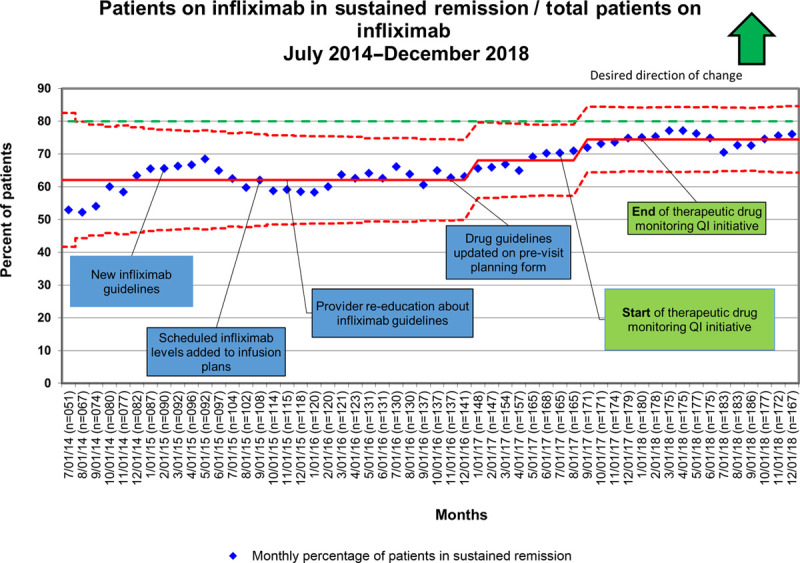
Infliximab patients in sustained remission. This P chart shows the % of patients receiving infliximab in sustained remission from July 2015 to August 2019 as defined by the ICN PGA. The chart shows the monthly percentage of infliximab patients in sustained remission (blue dotted line), the average proportion of patients in sustained remission (solid red line), and the control limits that show process stability over time. Data call-outs highlight the beginning and end of the therapeutic drug monitoring QI initiative discussed.

Last, our initiative did impact our global aim of improving sustained clinical remission rates for IBD patients receiving infliximab. The initial average sustained remission rate was 62% during early data collection in 2016, which improved to 70% with the start of the therapeutic drug monitoring QI initiative. This improved to 75% by January of 2018 (Fig. 6). This rate of sustained clinical remission continued through December 2018, indicating lasting improvement.

## DISCUSSION

Using QI methodology, we successfully created, implemented, and refined a process to increase the number of IBD patients with therapeutic infliximab levels at any point in treatment at a large urban academic center. Achieving therapeutic infliximab levels is associated with improved remission rates and increased durability of drug response in pediatric IBD.^9,23^ This rise in the number of patients with optimal levels likely impacted our center’s improvement in sustained clinical remission rates in those receiving infliximab.

Our QI work mirrors and builds on what has been previously reported including a recent QI effort to increase the number of infliximab and adalimumab levels drawn on patients during the drug induction period.^24^ Our work shows that it is feasible to track and sustain drug levels during the induction, and throughout the maintenance phase of therapy. We also used standardized electronic alerts through the EMR to automatically remind providers to appropriately react to suboptimal drug troughs, a reliable decision support method to improve adherence. This type of intervention could be implemented at medical centers starting infusion plan integration into the EMR or those with established therapy plan processes. We also saw a substantial change in our process measures following physician education and personalized email reminders. These are simple interventions that can be done even without EMR integration.

Despite the improvements shown, we understand that this project has limitations. For example, patients receiving infliximab outside of one of our CCHMC locations were excluded due to the inability to reliably track infusion occurrences and reliably obtain and review drug levels due to lack of integration in our EMR. Our program has since started an additional QI initiative focused on the tracking and management of external infusion patients, given the need to integrate these patients into our care algorithm. Furthermore, we did not track barriers to achieving a 100% success rate in our process measure of adherence to our infliximab care algorithm. It is possible that provider or patient preference continued to play a role. There was no marked change in adherence to the infliximab care algorithm after the rollout of the patient/family educational materials, but this was an important tool for patient and family education. We also speculate that third-party payers may have denied drug levels or dose changes based on proactive therapeutic drug monitoring. Furthermore, we are continuing to manually track data on our process measures to determine if the BPA alone is sufficient to sustain compliance with the recommended algorithm. Although we saw a temporal association between improved adherence to our infliximab care algorithm and increased sustained clinical remission rates, we did not analyze other clinical practice or patient population variables that may have also positively impacted our sustained clinical remission rate for those receiving infliximab during this period.

The success of our QI initiative demonstrates that a project of this scale can be implemented even within a large academic hospital with a multitude of physicians with varying areas of expertise and different patient panels. This process could similarly be adapted to standardize the monitoring of other medications or adherence to standard care algorithms. Given the success of our QI efforts in proactive therapeutic drug monitoring in IBD patients on infliximab, we are optimistic that expansion of this work to include patients on other biologic therapy will continue to impact our global aim of improving sustained clinical remission rates in children and young adults with IBD.

## DISCLOSURE

The authors have no financial interest to declare in relation to the content of this article.

## Supplementary Material


