# Supplementary figures and images for: Quality Improvement Methodology Optimizes Infliximab Levels in Pediatric Patients with Inflammatory Bowel Disease
Source: Pediatr Qual Saf. 2021 May 5;6(3):e400. doi: 10.1097/pq9.0000000000000400 (PMC8104299; doi:10.1097/pq9.0000000000000400)

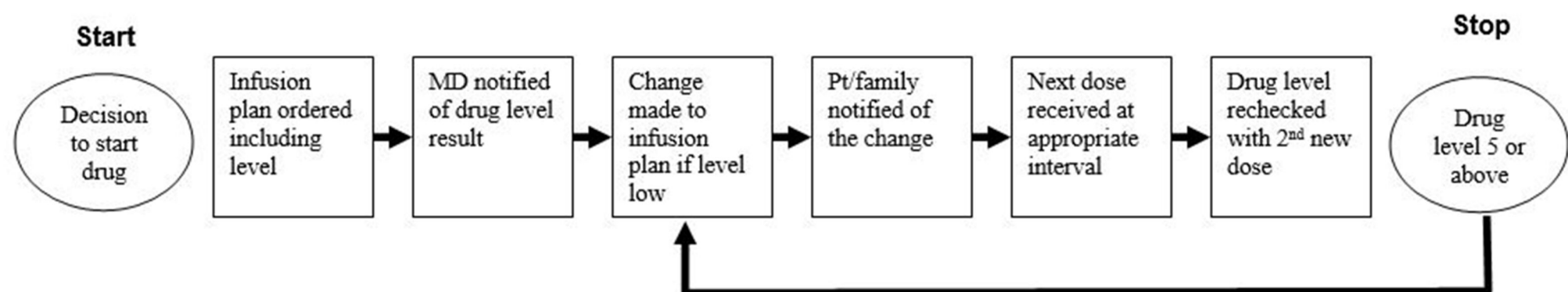

Supplement: Supplementary file 1 [file pqs-6-e400-s001.pdf]

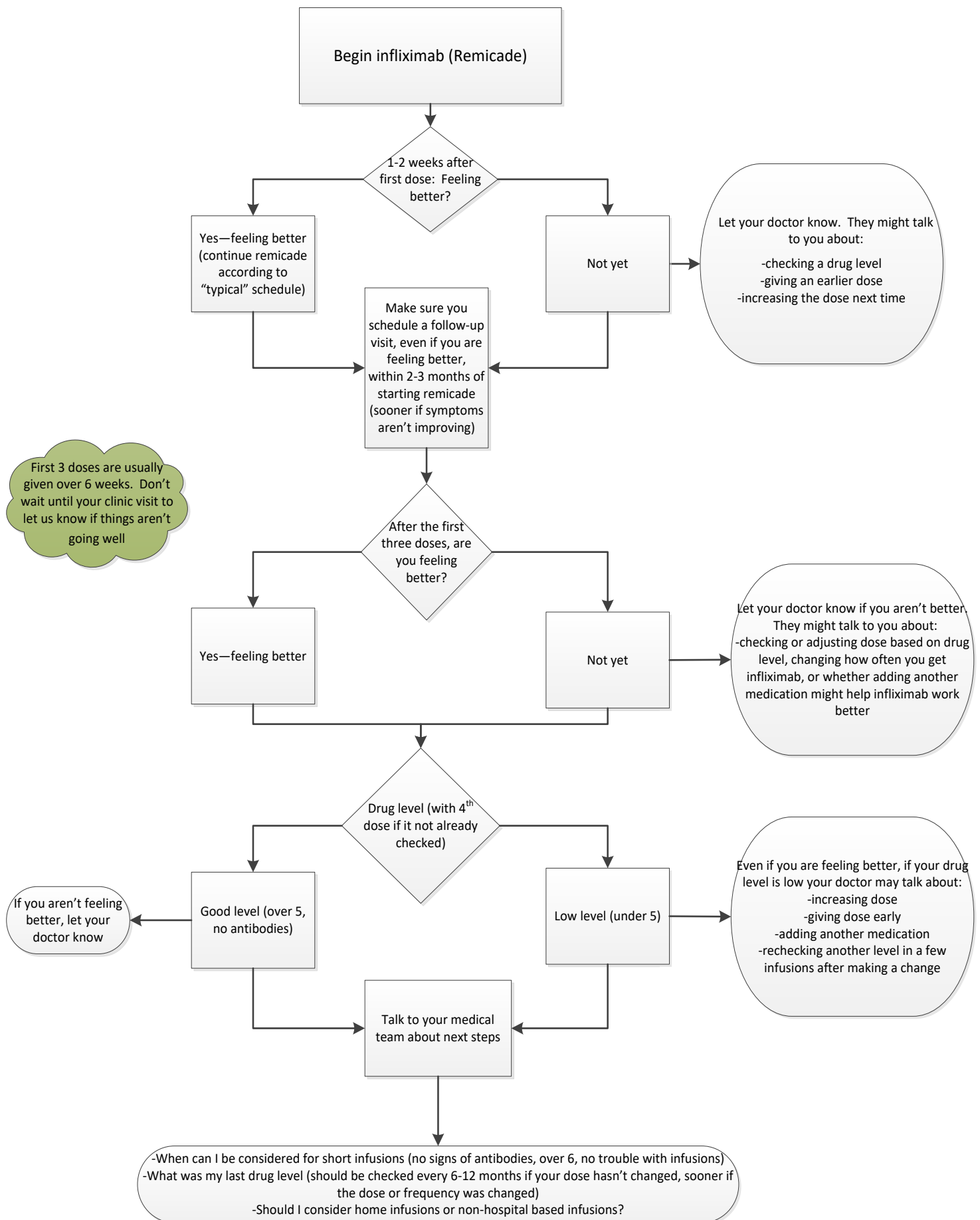

Supplement: Supplementary file 2 [file pqs-6-e400-s002.pdf]
